# Renal Cell Carcinoma of Native Kidneys in Kidney Allograft Recipients: Are There Any Guidelines for Management?

**DOI:** 10.3390/jcm15124478

**Published:** 2026-06-10

**Authors:** Letycja Róg, Michał Pyrża, Ewa Wojtaszek, Tomasz Głogowski, Aleksandra Kaszyńska, Zuhier Shebani, Leszek Kraj, Vadym Matsibora, Jolanta Małyszko

**Affiliations:** 1 Department of Oncology, Medical University of Warsaw, ul. Banacha 1A, 02-097 Warsaw, Poland; letycja.rog@uckwum.pl (L.R.); leszek.kraj@wum.edu.pl (L.K.); 2 Department of Nephrology, Dialysis, and Internal Medicine, Medical University of Warsaw, ul. Banacha 1A, 02-097 Warsaw, Poland; michal.pyrza@uckwum.pl (M.P.); ewa.wojtaszek@wum.edu.pl (E.W.); tomasz.glogowski@wum.edu.pl (T.G.); aleksandra.kaszynska@wum.edu.pl (A.K.); zuhier.shebani@uckwum.pl (Z.S.); 3 2nd Department of Clinical Radiology, Medical University of Warsaw, 02-097 Warsaw, Poland; vadym.matsibora@uckwum.pl

**Keywords:** renal cell carcinoma, kidney transplantation, kidney transplant recipients, ultrasound examinations

## Abstract

**Background**: Renal cell carcinoma (RCC) accounts for nearly 90% of kidney cancers. Transplantation is the best treatment for kidney failure, associated with improved survival, quality of life, and lower societal costs compared with remaining on dialysis. Thanks to modern immunosuppression, rejection rates have decreased. Cancer is the second most common cause of morbidity and mortality in kidney transplant recipients (KTRs) after cardiovascular disease. KTRs are at increased cancer risk due to chronic immunosuppression. **Case report**: We report a 54-year-old kidney transplant recipient without prior history of malignancy who developed metachronous bilateral RCC early posttransplant (first RCC within 3 months after kidney transplantation and second RCC after one year later). Both tumours were treated with nephrectomy. **Conclusions**: Early diagnosis enabled appropriate oncologic management while preserving graft function. It should also be stressed that beside graft assessment, abdominal sonography should not be forgotten in kidney allograft recipients, in particular, in certain high-risk patients (i.e., elderly, male, with longer dialysis vintage, smokers, obese, with high burden of immunosuppression including pretransplant immunosuppressive therapy, induction at transplantation, etc.).

## 1. Introduction

Renal cancer is the 14th most common malignancy worldwide, with >430,000 new cases diagnosed in 2020. The incidence varies geographically, with a higher incidence in Europe and North America. Renal cell carcinoma (RCC) accounts for ∼90% of all renal cancers [1].

Due to the increasing occurrence of RCC in the general population and the high prevalence of chronic kidney disease among cancer patients, many people with a previous RCC may eventually require renal replacement therapy including kidney transplantation [2].

Kidney transplantation is widely regarded as the best treatment option for patients with kidney failure. Compared with remaining waitlisted in dialysis, kidney transplantation is associated with improved survival and quality of life and entails a lower cost for society. Modern immunosuppression has reduced acute rejections to <10%, and >90–95% of grafts function beyond the first year [3]. In parallel, mortality has decreased, with 60–80% of patients surviving > 10 years after a first deceased or living donor kidney transplant [3,4].

Cancer is the second most common cause of mortality and morbidity in kidney transplant recipients after cardiovascular disease. Kidney transplant recipients have at least a twofold higher risk of developing cancer or dying from it than the general population. The increased risk of de novo and recurrent cancer in transplant recipients is multifactorial and attributed to oncogenic viruses, immunosuppression and altered T cell immunity [5,6,7].

Furthermore, compared with the general population, recipients of kidney transplants have a higher risk (up to seven-fold) of RCC, mainly due to long-term immunosuppression preventing graft rejection [7,8]. RCC accounts for 80–90% of urologic cancers in KTRs [9,10,11]. Due to increased abdominal imaging, most kidney masses detected in kidney transplant recipients are typically early, low-grade, small kidney masses, of which 75–80% are RCC, with the risk of metastasis at presentation being <2%. Moreover, 90% of RCCs develop in the native kidneys as opposed to the allograft. Risk factors for the development of RCC post-transplantation includes male sex [5,6,7], age at transplantation, obesity, smoking, and acquired cystic kidney disease (ACKD) [12,13,14], white ethnicity and extended time on dialysis before transplantation [5,6,7]. Hickman et al. [15] further emphasized dialysis duration ≥ 3 years, glomerular disease, and hypertensive or vascular nephropathy as risks.

The cancer can be encountered at different steps in the transplant process. RCC found during work-up of a transplant candidate needs treatment, and to limit the risk of recurrence, a mandatory observation period before transplantation is usually recommended [5].

Finally, it is also worth mentioning that in patients with a history of cancer before transplantation, the risk of disease recurrence is generally low, with <10% of patients developing recurrent cancer or a second new cancer after transplantation [7].

However, when RCC is diagnosed posttransplant, treatment in KTRs poses challenges regarding adjustments to immunosuppression and oncologic treatments, as well as a high risk of drug–drug interactions. Moreover, RCC post-radiotherapy can adversely affect the allograft function and long-term survival [5,6,7].

## 2. Clinical Case Description

**Figure 1:**
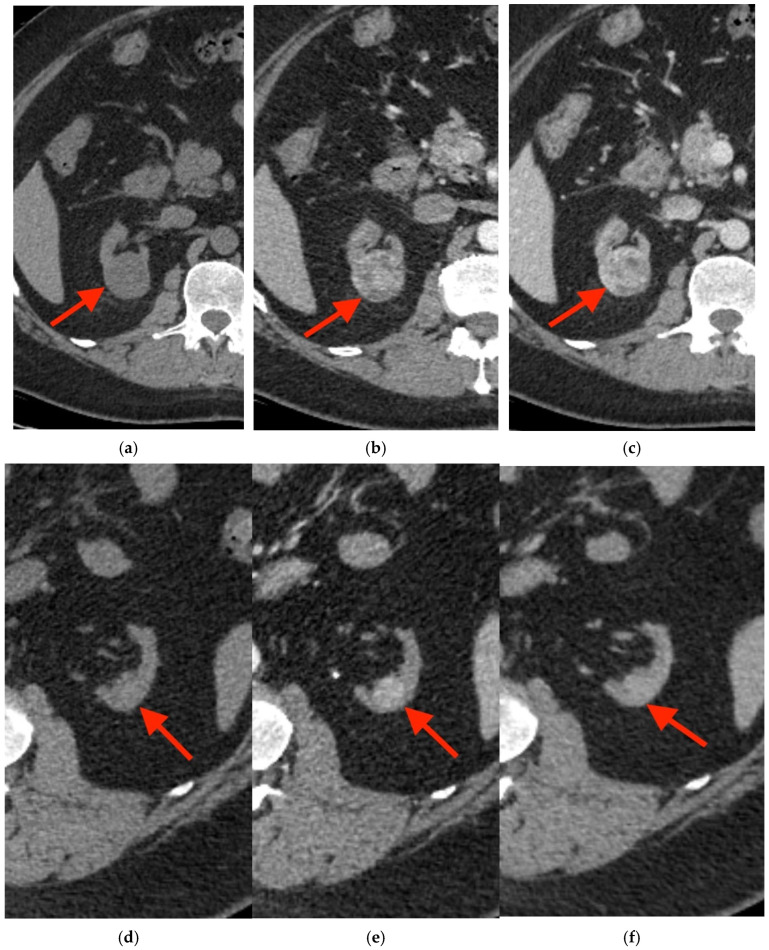
(**a**–**f**) Bilateral renal masses demonstrate heterogeneous contrast enhancement and marked hypervascularity in the arterial phase. In the venous phase, a washout pattern is observed in both the right- and left-sided lesions. The right renal tumour, measuring 32 × 32 mm, is in the posterior segment of the right kidney. The left renal lesion, measuring 16 mm, is also situated in the posterior segment of the left kidney. (**a**) Right kidney, native phase. (**b**) Right kidney, arterial phase. (**c**) Right kidney, venous phase. (**d**) Left kidney, native phase. (**e**) Left kidney, arterial phase. (**f**) Left kidney, venous phase. Arrows show renal massRCC.

A 54-year-old man with an arteriovenous fistula on the left forearm (created on 6 June 2020) who has been under the care of the Dialysis Unit at a regional hospital for more than two years (since 11 January 2020) due to end-stage renal failure and other numerous internal diseases, including arterial hypertension complicated by hypertensive neuroretinopathy, secondary hyperparathyroidism, chronic gastritis due to Helicobacter pylori infection, prostatic hyperplasia, chronic venous insufficiency, hypercholesterolemia and obesity (BMI 31.7 kg/m^2^). He had no immunosuppressive therapy before and during hemodialysis treatment. The patient underwent transplantation of an allogeneic left kidney from a deceased donor to the right iliac fossa (26 November 2021) in our hospital with immunosuppressive protocol including basiliximab as an induction therapy (cold ischemia time more than 24 h), together with tacrolimus, mycophenolate mofetil-MMF and steroids. At that time the patient had residual diuresis of around 500 mL per day. The perioperative and postoperative periods were uneventful. When the observation was completed, the patient was discharged home with a standard immunosuppressive regimen consisting of tacrolimus, MMF and prednisone. However, on 10 December 2021, the patient was admitted to the Surgery Department of our hospital on an emergency basis due to wound dehiscence after kidney transplantation to the right iliac fossa. The wound was sutured, and after observation the patient was discharged home. Nevertheless, throughout this time, the patient maintained satisfactory diuresis. Approximately one month after hospitalization, in mid-January 2022, the patient was referred from the transplant outpatient clinic after a routine check-up visit due to abnormalities in control laboratory tests (elevated serum creatinine and neutropenia). In the interview it was noted that for about a week the patient had observed a deterioration in appetite, weakness, and loose bowel movements—he denied fever and cold symptoms. A complete set of laboratory tests was performed, and reactivation of CMV infection was diagnosed (111,000 of copies/mL). Ganciclovir treatment was started at a dose adjusted to the graft function and MMF was withdrawn. After that, the number of copies decreased to 1280/mL, the number of leukocytes normalized, the clinical symptoms disappeared, and the graft function improved. During hospitalization, a routine abdominal ultrasound was also performed in which a lesion in the upper pole of the native right kidney was observed (the registry showed the pre-transplant ultrasound was normal). In computed tomography the lesion was described as most likely corresponding to RCC (32 × 32 mm) with enlarged lymph nodes at the level of the tumour (Figure 1a–c). After qualification for the procedure, right nephrectomy due to RCC was performed in March 2022 (four months after transplantation). In July of the same year, the patient was hospitalized again due to another reactivation of CMV (3000 copies/mL). At the beginning of March 2023, the patient was undergoing treatment for CMV reactivation. The patient was admitted to our clinic due to a suboptimal graft function—a clinical suspicion of stenosis of the transplanted renal artery. In imaging studies, abdominal ultrasound stenosis was excluded, but a focal lesion (most likely RCC) was found in the left native kidney (as detailed in the CT imaging in Figure 1d–f, illustrating the small mass). During hospitalization, hypertensive and diuretic treatment was modified, resulting in clinical improvement but not a full restoration of the graft function of the transplanted kidney. Other causes of deterioration of the graft function were excluded (BKV, EBV negative, CMV with a decreasing copy number). Therefore, graft biopsy on indication was considered. However, it was postponed for several days due to antiplatelet treatment. In addition, during the surgical and radiological consultation for renal mass in the left native kidney, the patient qualified for left-sided nephrectomy. On 5 April 2023, a biopsy of the transplanted kidney was performed, and 12 days later, the histology result was obtained, which did not show any signs of acute rejection. The results of immunomorphological tests revealed fibrillary glomerulosclerosis, focal segmental glomerulosclerosis, most likely secondary, and minor foci of stromal fibrosis and tubular atrophy. In addition, as previously mentioned, the patient was qualified and prepared for nephrectomy of the left native kidney. The surgery was performed on 27 April 2023, and the postoperative period was uneventful. Histopathology of the specimen from the first tumour showed clear cell renal cell carcinoma G1 pT1a, Nx, R0, and in the subsequent specimen from the second tumour, multilocular cystic renal neoplasm of low malignant potential pT1a, Nx, R0 was described.

In December of the same year, the patient was hospitalized again to complete oncological diagnosis due to the presence of fibrillary glomerulopathy in the transplanted kidney. An abdominal CT scan showed a lesion in the bed after the left kidney had been removed. On 7 December 2023, a core needle biopsy of the lesion described in the imaging study was performed under CT guidance (histopathology did not reveal any neoplastic tissue). In addition, control endoscopic examinations were performed. A sigmoid polyp, which was hyperplastic in the microscopic image, was removed.

The patient was all the time under strict oncological supervision. He underwent periodic imaging assessments. One of these assessments revealed a stable appearance of the lesion in the left nephrectomy bed; however, it also noted an increase in the size of pulmonary nodules. The patient was subsequently admitted for a scheduled CT-guided biopsy of the lung nodules at the end of October 2024. The procedure was completed without any complications; however, the biopsy results were non-diagnostic (in the submitted material only skeletal muscle fibres were described). That is why, a month after the result was obtained, the patient was readmitted to the clinic for a repeated CT scan of the chest. At that time the image of the lung nodules was stable. The consulting pulmonologist recommended observation without the need to repeat the diagnostic biopsy. The patient remains under the care of our department and is under strict oncological supervision (Table 1).

## 3. Discussion

In our case, the risk factors for RCC of male sex, obesity, ESRD due to hypertensive nephropathy, and dialysis (though < 2 years) were present. Sonography during evaluation was unremarkable; thus, no further imaging was performed. Donor-related risks, including older age, hereditary renal disease, or cancer history [16], were absent in our case. Data on the RCC posttransplant are limited (Table S1 in Supplementary Materials). As reported by Schmidt et al. [17], KTRs had a higher risk of RCC and presented at a localized stage with comparable overall survival rates to non-transplant patients with RCC. In the retrospective study from Romania, with an RCC incidence rate of 0.78% (18 patients out of 2283 KTRs who underwent transplantation between 2008 and 2023) [18], more that 50% of renal tumours were diagnosed incidentally during routine posttransplant follow-up, supporting the potential value of structured screening programmes. In the recent case report on bilateral RCC, diagnosis was made 14 months posttransplant at routine ultrasonography of the native kidneys [19], whereas Araibi [20] described a case of synchronous bilateral native kidneys papillary RCC, discovered by serendipity on CT 10 yeast posttransplant.

Tao et al. [21] presented three KTRs who developed native kidney RCC 6–15 years post-transplantation, whereas in our case first RCC was diagnosed within 3 months after transplantation and second RCC after one-year posttransplant. The authors underscored the significant risk posed by RCC, particularly in patients who omit regular imaging surveillance; however, they did not define standard posttransplant imaging. In addition, they also stressed that ultrasonography was the preferred screening modality due to its simplicity, non-invasiveness, and high resolution, and was particularly suitable for evaluating solid or cystic renal masses in native or grafted kidneys. They also advocate considering CEUS (contrast-enhanced ultrasonography) in differential diagnosis of pseudocapsules and cystic renal lesions compared to contrast-enhanced CT [22]. CEUS spares the use of a nephrotoxic agent, in the case of suboptimal graft function, and is accurate in the characterization of small renal masses and enhancement patterns [22]. Additionally, CEUS provides information about dynamic perfusion due to real-time monitoring of contrast agent inflow and outflow within renal lesions. We do not use CEUS on an everyday basis in our hospital.

There is no consensus on optimal RCC screening in KTRs [23,24,25,26,27,28]; see Table S2 in Supplementary Materials. This does not help practicing physicians taking care of kidney transplant recipients in the logistics of posttransplant care with regard to malignancy screening. Posttransplant care depends upon the organization of the healthcare system, reimbursement issues, and available standards/recommendations or clinical practice in each country or even each centre.

Previously, we advocated annual abdominal ultrasound, dermatological exams, and optimal immunosuppression for high-risk KTRs [29], noting insufficient evidence and cost-effectiveness analyses for firm guidelines (timing, imaging method, population). Similar conclusions have been drawn by others [30,31]. In a systematic scoping review of 42 case reports and 11 retrospective cohorts (274 RCC cases in grafts), Tanariyakul et al. highlighted the substantial prevalence of asymptomatic RCC and the need for rigorous surveillance [32].

In the most recent review, we not only presented the epidemiology of malignancy after transplantation, diagnosis, treatment, and screening, but we stated the limitations of the published data, proposed research priorities and acknowledged gaps and unmet needs [33].

Pommerolle et al. [34] suggested that modification of immunosuppressive treatment should be discussed in patients with RCC, as we did with our patient. However, detailed information on immunosuppressive regimens is not commonly found in reported cases, nor do they discuss its modification at time of cancer diagnosis and subsequent treatment.

As malignancies are the third most common cause of death in kidney transplant recipients and urinary tract cancers have become the third most common malignancy after kidney transplantation, real-world data on the diagnosis and management of RCC after transplantation are of paramount importance.

As current recommendations on screening and therapy are still largely based on data from the general population, their validity in kidney transplant recipients remains uncertain; therefore, gathering more real-world data to develop recommendations for this vulnerable population is an unmet need and requires worldwide collaboration.

Despite the increasing recognition of RCC risk among kidney transplant recipients, there is still no universal consensus regarding routine RCC screening strategies. Current recommendations remain heterogeneous and are largely influenced by differences in healthcare systems, organization of posttransplant care, and the availability of imaging, as well as local clinical practice.

## 4. Conclusions

The educational value of the presented case is raised awareness that even in early posttransplant periods, RCC could be diagnosed in patients with completely normal abdominal sonography up to 6 months before transplantation. Moreover, one year after first nephrectomy, the second RCC was diagnosed in our patient. It should also be stressed that despite suboptimal graft function we did not hesitate to perform CT with contrast to ensure adequate diagnosis and enable timely and appropriate management to improve long-term outcomes. Moreover, this is a challenging case, with early RCC diagnosis requiring reduction in immunosuppression to balance the risk of acute rejection. However, no causal inference can be made from a single case. It should also be stressed that beside graft assessment, abdominal sonography should not be forgotten in kidney allograft recipients, in particular, in certain high-risk patients (i.e., elderly, male, with longer dialysis vintage, smokers, obese, with high burden of immunosuppression including pretransplant immunosuppressive therapy, induction at transplantation, etc.).

Managing RCC in KTRs requires a careful adjustment of immunosuppression and attention to drug–drug interactions. Our findings highlight the need for tailored screening protocols that enable early detection and timely intervention, significantly improving outcomes. Future research should focus on evidence-based guidelines for RCC surveillance and management in the transplant population, ensuring optimal care addressing both renal and oncological health.

## Figures and Tables

**Table 1:** Chronological timeline of patient’ clinical course.

Date/Period	Clinical Course
January 11, 2020	The patient started maintenance hemodialysis due to end-stage renal failure and was under care of hospital dialysis unit for chronic hemodialysis program.
June 6, 2020	Creation of an arteriovenous fistula on the left forearm.
November 26, 2021	Deceased-donor kidney transplantation was performed with implantation of the allogeneic left kidney into the right iliac fossa.
December 10, 2021	Emergency admission to the Surgical Department due to wound dehiscence after kidney transplantation. Surgical wound closure was performed, and the patient was discharged after observation.
Mid-January 2022	The patient was referred from the transplant outpatient clinic because of elevated serum creatinine concentration and neutropenia detected during routine follow-up laboratory tests.
January 2022	Reactivation of CMV infection was diagnosed. MMF was withdrawn and ganciclovir therapy adjusted to graft function was initiated.
January–February 2022	Clinical and laboratory improvement was achieved: normalization of leukocyte count and improvement of graft function. A lesion in the upper pole of the native right kidney on sonography was found.
February 2022	Computed tomography demonstrated a lesion measuring 32 × 32 mm in the native right kidney, highly suggestive of RCC, with enlarged lymph nodes adjacent to the tumour.
March 2022	Right-sided nephrectomy due to RCC of the native kidney
July 2022	Rehospitalization due to another CMV reactivation.
Early March 2023	Hospitalization due to suboptimal graft function and clinical suspicion of transplant renal artery stenosis.
March 2023	Transplant renal artery stenosis was excluded, and focal lesion in the native left kidney was found, most likely corresponding to RCC.
March–April 2023	Other causes of graft dysfunction were excluded (negative BKV and EBV results, decreasing CMV viral load). Evaluation for left native nephrectomy.
April 5, 2023	Biopsy of the transplanted kidney.
April 17, 2023	No evidence of acute rejection; fibrillary glomerulosclerosis, most likely secondary focal segmental glomerulosclerosis, and minor foci of stromal fibrosis and tubular atrophy.
April 27, 2023	Left-sided nephrectomy of the native kidney.
December 7, 2023	Abdominal CT revealed a lesion within the left nephrectomy bed. CT-guided core needle biopsy was performed and showed no evidence of neoplastic tissue.
December 2023	Follow-up endoscopic examinations were performed. A sigmoid polyp was removed, and histopathological examination confirmed a hyperplastic lesion.
End of October 2024	Follow-up imaging demonstrated stable appearance of the lesion within the left nephrectomy bed but enlargement of pulmonary nodules. CT-guided biopsy of the pulmonary nodules was performed without complications; however, the result was non-diagnostic and showed only skeletal muscle fibres.
November 2024	On-chest CT pulmonary nodules remained stable, and the consulting pulmonologist recommended further observation without repeated biopsy.
Present time	The patient remains under the care of the transplant centre and continues strict oncological surveillance.

## Data Availability

The data presented in this study are available on request from the corresponding author due to privacy.

## Supplementary Materials

The following supporting information can be downloaded at: https://www.mdpi.com/article/10.3390/jcm15124478/s1, Table S1. Studies on renal cell cancer in kidney transplant recipients; Table S2. Renal cell cancer screening according to available guidelines.
