# Supplementary material for: Renal Cell Carcinoma of Native Kidneys in Kidney Allograft Recipients: Are There Any Guidelines for Management?
Source: J Clin Med. 2026 Jun 10;15(12):4478. doi: 10.3390/jcm15124478 (PMC13302036; doi:10.3390/jcm15124478)
Supplement: Supplementary file 1 [file jcm-15-04478-s001.zip › jcm-4326851-supplementary.pdf]

Table S1 Studies on renal cel cancer in kidney transplant recipients

Source	Study Type	Population	Screening/Imaging Recommendation	Suggested Frequency	Key Findings
Schmidt et al. [17]	Retrospective study	4012 KTRs	No specific screening protocol proposed	Not specified	KTRs had a higher risk of RCC and were more frequently diagnosed at a localized stage compared with non-transplant RCC patients.
Moldoveanu et al. [18]	Retrospective cohort	2283 KTRs	Routine post-transplant surveillance imaging	Not specified	More than 50% of RCC cases were diagnosed incidentally during routine follow-up, supporting structured screening programs.
Gautam et al. [19]	Case report	KTRs with bilateral RCC	Routine ultrasonography of native kidneys	Diagnosis at 14 months post-transplant during scheduled US examination	RCC detected asymptomatically on surveillance imaging.
Araibi H. [20]	Case report	KTRs with bilateral papillary RCC	CT imaging	Not specified	RCC identified incidentally on CT 10 years after transplantation.
Tao et al. [21]	Case series (3 KTRs)	KTRs with native kidney RCC	Regular imaging surveillance recommended	Not specified	RCC occurred 6–15 years post-transplant; omission of imaging surveillance was highlighted as a risk factor for delayed diagnosis.
Tao et al. [21]	Expert opinion within case series	KTRs	Ultrasonography preferred as first-line screening modality	Not specified	US considered simple, non-invasive, and suitable for detecting solid and cystic renal lesions in native and transplanted kidneys.
He M et al. [22]	Diagnostic imaging study/review	Patients with renal lesions	Contrast-enhanced ultrasound (CEUS) for characterization of renal masses	When lesions are detected	CEUS may improve characterization of small renal masses and cystic lesions while avoiding nephrotoxic contrast agents.
Our previous recommendations [29]	Expert opinion	High-risk KTRs	Abdominal ultrasonography	Annually	Annual abdominal US proposed for RCC surveillance in high-risk KTRs.
Tanariyakul et al. [32]	Systematic scoping review	42 case reports and 11 retrospective cohorts (274 RCC cases)	Rigorous surveillance advocated	Not specified	High prevalence of asymptomatic RCC supports systematic surveillance strategies.

Table S2 Renal cell cancer screening according to available guidelines

**Source**	**Study Type**	**Population**	**Screening/Imaging Recommendation**	**Suggested Frequency**	**Key Findings**
KDIGO 2009 [24]	International clinical practice guideline	Kidney transplant recipients	Routine screening for RCC is not recommended in asymptomatic kidney transplant recipients.	Not specified	The guideline found no evidence demonstrating a survival benefit from systematic RCC screening in the general transplant population.
European Association of Urologists [26]	Guideline recommendation	Kidney transplant recipients	Regular surveillance of native kidneys is recommended due to the increased incidence of RCC after transplantation.	Annually	Annual screening was advised, particularly because RCC is frequently diagnosed in native kidneys and may remain asymptomatic until advanced stages.
ERBP [27]	European expert guideline	Kidney transplant recipients	Ultrasonographic screening of native kidneys is recommended, particularly in recipients at increased risk of RCC.	Not specified	The guideline supported the use of ultrasound as a non-invasive surveillance tool but did not define an optimal screening interval.
KDIGO 2020 [28]	Clinical practice guideline for transplant candidates	High-risk kidney transplant candidates	Renal ultrasonography is recommended before transplantation in candidates at increased risk of RCC, including those with ≥3 years of dialysis exposure, acquired cystic kidney disease, family history of RCC, or analgesic nephropathy.	Baseline evaluation before transplantation; no post-transplant interval specified	The recommendation highlights targeted screening of high-risk individuals; however, the strength of recommendation and quality of evidence were not formally graded.
